# Organ Donation Awareness among Family Members of ICU Patients

**DOI:** 10.3390/medicina59111966

**Published:** 2023-11-08

**Authors:** Petru Cotrău, Marcel Negrău, Viviana Hodoșan, Adriana Vladu, Cristian Marius Daina, Dorel Dulău, Carmen Pantiș, Lucia Georgeta Daina

**Affiliations:** 1 Faculty of Medicine and Pharmacy, Doctoral School of Biomedical Sciences, University of Oradea, 1 University Street, 410087 Oradea, Romania; pinta.vivi@yahoo.com (V.H.); adrianavladu68@yahoo.com (A.V.); doreldulau77@gmail.com (D.D.); 2 Emergency Clinical County Hospital of Oradea, 410169 Oradea, Romania; negrau.marcel@gmail.com (M.N.); cristi_daina@yahoo.co.uk (C.M.D.); pantisc@yahoo.com (C.P.); lucidaina@gmail.com (L.G.D.); 3 Department of Surgical Disciplines, Faculty of Medicine and Pharmacy, University of Oradea, 1 University Street, 410087 Oradea, Romania; 4 Psycho-Neurosciences and Recovery Department, Faculty of Medicine and Pharmacy, University of Oradea, 1 University Street, 410097 Oradea, Romania

**Keywords:** organ donation, brain death, willingness to donate, ICU patient’s relatives

## Abstract

*Background and Objectives*: With one of the lowest donation rates in the European Union, Romania faces challenges in organ donation from brain death donors, within an opt-in system. This study aims to measure the attitudes and knowledge of ICU patient’s relatives toward organ donation. *Materials and Methods*: A descriptive cross-sectional study was conducted in the intensive care unit of the Emergency Clinical County Hospital of Oradea, Romania. A 24-item self-administered questionnaire (*N* = 251) was used to collect data on knowledge about organ and tissue donation and transplantation, as well as the willingness to donate. *Results*: A high degree of awareness and willingness for organ donation and transplantation was recorded. The main positive predictor of willingness to donate was the perception of helping others by donating their organs after brain death (β = 0.537, OR = 1.711, *p* < 0.05), and the main negative predictor was the idea that the whole body should be buried intact (β = −0.979, OR = 0.376, *p* < 0.01). *Conclusions*: A basic understanding of organ donation and transplantation and favorable attitudes toward organ donation were registered. Families’ interviews for organ donation consent may be affected due to extreme emotional distress.

## 1. Introduction

It is well known that organ transplantation saves lives and improves patients’ quality of life with end-stage organ failure [1]. The demand for organ donors is constantly increasing. The gap between the number of patients on waiting lists and the number of organ donors has increased in recent decades, leading to an increase in the shortage of transplantable organs. Organ shortages have become a significant policy issue in countries worldwide [2].

Recognized as a selfless and widely praised act [3], a long-standing challenge in increasing the organ donation rate from brain death donors is represented by the refusal of families to donate [4,5]. Even if it is unlikely that demand could ever be met, the growing need for organ donation has led to different approaches and methods in obtaining consent for donation: informed consent and presumed consent [6].

Defined as optional registration, opt-in or informed consent requires individuals to take an explicit affirmative action to become a donor by registering their will during their lifetime in the donor registry and the informed consent from the donor’s family [7]. Opt-out or presumed consent considers all individuals residing in a country as potential deceased organ donors unless they specifically express their intention not to be considered donors [8,9]. Presumed consent has been considered in several studies as a possible way to increase the availability of transplantable organs; however, the presumed consent system alone is unlikely to explain variation in organ donation rates [10]. Instead, different factors are involved: legislation, transplant system, infrastructure, social attitudes, and intervening variables (population general medical knowledge, educational level, social and cultural aspects, and religious views on organ donation) [11,12].

Of the 27 states of the European Union, Romania, together with six other member states, have an opt-in consent system, 20 states have an opt-out consent system, and one state has a mixed system [13]. The National Transplant Agency coordinates organ donation and transplant activity in Romania through the six regional centers and 33 institutions under its control. With a population of approximately 19 million people, from 2013 to 2021, Romania registered an organ donation rate of 4.7 (per million population), one of the lowest in the European Union [14,15,16,17,18]. From 2018 to 2021, the total number of national family interviews for organ donation in brain death donors was 829, with a family refusal rate of 22.5% [19,20,21,22].

## 2. Materials and Methods

### 2.1. Participants and Procedure

We conducted a cross-sectional descriptive study in the intensive care unit of the Emergency Clinical County Hospital of Oradea, Romania, a level 1 ICU (this level includes all complex patients). We used convenience sampling and gathered data between November 2022 and February 2023. We chose to conduct our study in the ICU because it is where the diagnosis of brain death is confirmed and where discussions about organ donation consent are held. Convenience sampling was preferred to avoid additional psychological distress for the family members of ICU patients, as well as for ease of research and data availability. A total of 251 family members with relatives hospitalized in the ICU responded to our questionnaire (one adult family member per patient). None of the respondents had their relatives declared brain dead organ donors. All participants gave voluntary informed consent, and confidentiality was assured.

### 2.2. Instruments

We developed a 24-item self-administered questionnaire on previous researches [23,24,25,26,27] to collect data on knowledge about organ and tissue donation and transplantation, as well as the willingness to donate. Our questionnaire was distributed on paper and divided into four sections corresponding to the following: general knowledge about organ and tissue donation and transplantation (Q1–Q5, yes/no answers), willingness to donate (Q6–Q10, yes/no answers), attitudes toward organ donation (Q11–Q20, 5-point Likert scale), and sociodemographic variables (age, gender, educational level, religion).

### 2.3. Statistical Analysis

We conducted descriptive and inferential statistical analyses in IBM SPSS Statistics 29 software. Sociodemographic data were summarized as counts and percentages, and responses between groups were compared using Chi-square. Chronbach α coefficient was used to determine the internal validity and reliability of the 5-point Likert scale by measuring attitudes toward organ donation. A value of 0.708 was recorded. In addition, we performed multinomial logistic regression to assess which attitudes are predictors of the willingness to donate one’s organs. A *p* value < 0.05 was considered to be statistically significant.

## 3. Results

### 3.1. Sociodemographic Characteristics of Respondents

Table 1 shows the sociodemographic distribution of the participants in gender, age, educational level, and religion. Of the 251 participants, 77.5% were female, and 22.5% were men, with a mean age of 39 (range 20–60 years, SD 10.5). Of these, 80.6% had completed a tertiary educational level (ICED 4 or higher), and 19.5% had completed upper secondary education (ISCED 3). The most common religious identification was Orthodox Christianity (77.7%), followed by Protestantism (15.5%), Roman Catholicism (5.2%), Greek Catholicism, and Neo-Protestantism (<1%).

### 3.2. General Knowledge about Organ and Tissue Donation and Transplantation

Table 2 contains five questions to determine respondents’ general knowledge about organ donation and transplantation. Their responses indicate a high degree of awareness for the terms organ donation and organ transplantation (95.6% selected option yes, meaning they heard about it). When asked if there is an age limit for organ donation, 40.6% responded with yes, 47.8% with no, and 11.6% did not know what to reply. The last two questions referring to the term brain death registered a similar response rate; 49% heard about the term, while 47.8% responded correctly when asked if the diagnosis of brain death is equivalent to irreversible death; however, 52.1% responded incorrectly or did not know how to reply, from which, we can assume that families may misunderstand the definition and meaning of brain death.

### 3.3. Willingness to Donate

**Figure 1:**
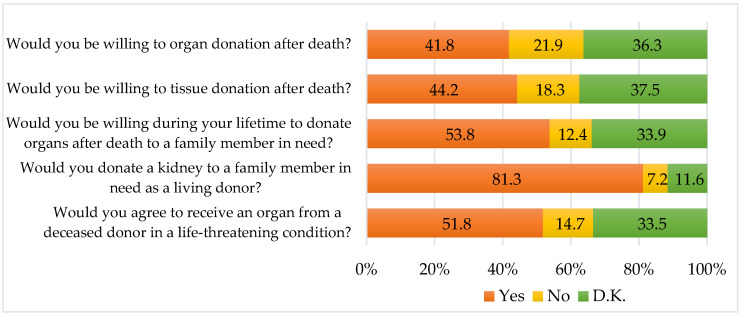
Respondents’ willingness to donate.

Figure 1 shows the percentages of respondents’ willingness for organ donation. Among 251 respondents, 41.8% expressed their willingness for organ donation after death, 44.2% expressed their willingness for tissue donation after death, 53.8% expressed their willingness for organ donation after death to a family member in need, 81,3% expressed their willingness to become a living kidney donor to a family member in need, and 51.8% expressed their agreement to receive an organ from a deceased donor in the case of a life-threatening condition.

A Pearson Chi-square was used to identify if there is a difference between different sociodemographic factors and willingness to donate (Table 3). Results of the Chi-square test showed a highly significant difference between age and willingness for organ (*χ*^2^ = 6.157, *p* < 0.046) and tissue donation (*χ*^2^ = 7.421, *p* < 0.024) and willingness to receive an organ or tissue (*χ*^2^ = 9.662, *p* < 0.001). Significantly, education (Q6: *χ*^2^ = 23.715, *p* < 0.001; Q7: *χ*^2^ = 36.257, *p* < 0.001; Q8: *χ*^2^ = 23.376, *p* < 0.001; Q10 *χ*^2^ = 16.758, *p* < 0.001) and religion (Q6: *χ*^2^ = 16.719, *p* < 0.002; Q7: *χ*^2^ = 12.748, *p* < 0.013; Q8: *χ*^2^ = 14.584, *p* < 0.006; Q10 *χ*^2^ = 24.901, *p* < 0.001) were found to be capable of influencing the willingness for organ donation, except for living donation.

### 3.4. Attitudes toward Organ Donation and Willingness to Donate

Table 4 presents respondents’ attitudes toward organ donation. Most of them present a generally positive attitude toward organ donation. A majority of our respondents strongly agree with the following statements: “I consider organ donation to be an altruistic gesture” (53.8%), “I like the idea of being able to help someone by donating organs after death” (51.4%), “I believe it is a moral duty to accept organ donation if you agree to receive organs” (45.0%), “My choice to become a donor reflects what my family feels I want” (43.8%), “Everyone should automatically be considered a potential donor” (31.1%), “After the age of 18, each individual should decide (legally register) whether or not they wish to become a potential organ donor” (47.4%), and “The family has the right to accept or not to donate organs in the case of brain death persons who during their lifetime did not have the opportunity to express their choice” (56.6%). On the other hand, 33.1% of our respondents somewhat disagree with the statement “Brain death patients who have not had the opportunity to express their choice during their lifetime should automatically be considered potential donors”, while 33.9% strongly disagree with the statements: “I consider it extremely important that my body be buried whole” and “I am afraid of “not being dead” in case of an organ procurement procedure” (33.5%).

The fidelity and reliability of the ten-item 5-point Likert scale were measured with Cronbach *α*. A value of 0.708 was recorded (Table 5). Based on the mean scores obtained, we found that respondents have favorable attitudes toward organ donation. The idea of being able to help someone by donating organs after death (mean 3.94, SD 1.261) and the perception of organ donation as a form of altruism (mean 3.84, SD 1.411) recorded the highest mean scores. At the opposite end, the importance of being buried intact (mean 2.39, SD 1.356) and the fear of “not being dead” in the case of an organ procurement procedure (mean 2.21, SD 1.137) recorded the lowermost mean scores.

We conducted a multinomial logistic regression analysis to predict respondents’ willingness to donate based on their attitudes toward organ donation (Table 6). Nagelkerke’s R-square value of 0.663 indicated a strong relationship between prediction and grouping. The Wald criterion demonstrated that: Q12—I like the idea of being able to help someone by donating organs after brain death (β = 0.537, OR = 1.711, *p* < 0.05), Q16—I consider it extremely important that my body be buried “whole” (β = −0.979, OR = 0.376, *p* < 0.01), Q17—Everyone should automatically be considered a potential donor (β = 0.789, OR = 2.202, *p* < 0.01), Q18—After age 18, each individual should decide (legally register) whether or not they wish to become a potential organ donor (β = −0.758, OR = 0.468, *p* < 0.05), Q19—Brain death patients who have not had the opportunity to express their choice during their lifetime should automatically be considered potential donors (β = 0.497, OR = 1.645, *p* < 0.05) were significant predictors.

## 4. Discussion

In an opt-in system, family members play a crucial role in the donation decision at the time of their beloved family member’s death. Denial and the misconception or misunderstanding of brain death [28], in addition to sociodemographic predictors, knowledge, and familial influences, are quoted in the literature [29,30,31,32,33] as predictors capable of influencing the decision to donate. An inadequate understanding of brain death and the presence of emotional distress can become a barrier for families [34,35,36] and even question the validity of the diagnosis [37]. Therefore, a clear understanding and explanation of the concept are necessary and can be amenable to education [38], as well as facilitate family decision making and increase the number of organs available for transplantation [39].

Most of our respondents (95.6%) are familiar with organ donation and transplantation, and most of them seem to be familiar with the diagnosis of brain death; however, 52.2% are not convinced that the diagnosis of brain death is equivalent to irreversible death. In the general perception, without basic medical knowledge, confusion may arise in understanding the definition of brain death in comparison to the traditional definition of biological death (irreversible cessation of circulatory and respiratory functions).

Different opinions and disagreements between family members can affect the donation process, which is considered one of the main barriers to giving consent [40]. These conflicts may arise due to personal views, beliefs, and attitudes toward organ donation, even if those views do not necessarily reflect the potential donor’s wishes or are unknown [41]. Another negative factor is considered to be religion; even if most major religions permit organ donation and transplantation, many religious members do not know this support exists [42,43,44], even though religious scholars have made efforts to promote organ donation [45]. These negative behaviors toward organ donation can be amended through public awareness and promotion of organ donation, alongside education and basic scientific knowledge about organ donation and procurement [46].

Respondents were willing to donate as living donors and deceased donors; however, age, educational level, and religious affiliation influence the willingness to donate. Multinomial logistic regression results show that the perception of helping others by donating their organs after brain death (*β* = 0.537, OR = 1.711, *p* < 0.05), the idea that everyone should be considered a potential donor (*β* = 0.789, OR = 2.202, *p* < 0.01), and those who have not decided during their lifetime should automatically be considered potential donors (*β* = 0.497, OR = 1.645, *p* < 0.05) were found to be significant positive predictors of willingness to donate. On the other hand, significant negative predictors were found to be the idea that the whole body should be buried intact in a traditional way (*β* = −0.979, OR = 0.376, *p* < 0.01) and the choice to legally register after the age of 18 whether or not to become a potential organ donor (*β* = −0.758, OR = 0.468, *p* < 0.05).

One of the 33 recognized facilities engaged in organ donation and transplantation is the Emergency Clinical County Hospital of Oradea, which assists in identifying possible brain death donors and ensures logistics control throughout the procurement stage of organ donation. In the Emergency Clinical County Hospital of Oradea, there were 96 family interviews for organ donation from 2018 to 2021, with a family refusal rate of 47.9%, which is higher than the national refusal rate for the same period (22.5%) [19,20,21,22]. Even though most participants in our survey showed a willingness to donate organs, there is still a high refusal rate for the ICU of the Emergency Clinical County Hospital of Oradea, with a rate of 47.9% from 2018 to 2021. It is difficult to identify the main reasons for this high refusal rate because, during this period, no research was conducted about organ donation awareness, knowledge, or attitudes in the ICU of the Emergency Clinical County Hospital of Oradea. From our findings, we can assume that an improper understanding of the concept of brain death can be interpreted as the main reason for this high refusal rate, taking into consideration that in our study, 52.1% responded incorrectly or did not know how to reply, when asked if the diagnosis of brain death is equivalent to irreversible death.

Organ donation is still considered a taboo subject in Romania. Bacușcă et al. conducted a study to identify positive predictors of organ donation in Romania. The authors showed that the majority of respondents had favorable attitudes toward organ donation; most of them were willing to donate if declared brain dead and were in favor of informed consent [47], individually as an expression of personal autonomy or through the family as next of kin [48]. In another study, which investigated through content analysis the Romanian online media coverage concerning organ donation, Petre and Băban came to the conclusion that a positive social media presence was established; however, communication is incoherent, which can lead to misconceptions and confusion [49]. This incoherence may be avoided through sustained campaigns to promote public awareness about organ donation by medical specialists through all available media channels.

In addition to the psychological distress for the family members of ICU patients, the lack of consent during a lifetime to become an organ donor leaves all responsibility on the family members who, in extreme emotional discomfort, must decide in this regard. An opt-out system removes this emotional discomfort bestowed on family members and would lead, according to empirical data [10,11], to higher organ donation rates, for example, in comparison with opt-in England, organ donation rates increased in Wales after adopting a soft opt-out system in 2015 [50].

Our study has several limitations. First, the sampling process was limited to ICU patient’s relatives, none of whom had their relatives declared brain dead organ donors. Another limitation is that we used a cross-sectional design, collecting data on attitudes and knowledge during the survey. Finally, longitudinal research could offer more information about the evolution of attitudes toward organ donation.

## 5. Conclusions

Our research is one of the few from Romania that focuses on organ donation awareness among family members of ICU patients. Our study showed a high level of awareness, positive attitudes toward organ donation, and willingness to become donors among participants.

An increased donation rate was associated with respondents with higher educational levels, and most of our respondents favored informed consent through the family decision process. The main positive predictor of willingness to donate was the perception of helping as a form of altruism, and the main negative predictor was the idea that the whole body should be buried intact.

In order to increase organ donation awareness amongst the general population, the inadequate understanding of brain death and various misconceptions must be addressed through awareness campaigns. In order to achieve maximum impact, these awareness campaigns should provide key messages sustained by scientific facts aimed at correcting misconceptions about the organ donation process. These targeted information campaigns should complement educational interventions because adequate information and education on organ donation and transplantation are needed to increase the willingness to become a donor.

Our findings may help Romanians become more aware of the value of organ donation and may be helpful information for policy making and future studies about knowledge, attitudes, and organ donation consent or refusal for a brain dead family member.

## Figures and Tables

**Table 1:** Sociodemographic characteristics.

Sociodemographic Variables		No.	%
Gender	Male	56	22.5%
	Female	193	77.5%
Age	20–30 years	69	27.5%
	31–40 years	86	34.3%
	41–50 years	65	25.9%
	51–60 years	31	12.4%
Educational level	ISCED 3 ^1^	49	19.5%
	ISCED 4 ^2^	20	8.0%
	ISCED 5–8 ^3^	182	72.6%
Religion	Orthodox Christianity	195	77.7%
	Roman Catholicism	13	5.2%
	Greek Catholicism	2	0.8%
	Protestantism	39	15.5%
	Neo-Protestantism Unaffiliated (Atheist/Agnostic)	2 /	0.8% /

^1^ Upper secondary education. ^2^ Tertiary non-university education (post-secondary education). ^3^ Bachelor studies, Master studies, Ph.D. studies.

**Table 2:** General knowledge about organ donation and transplantation.

Question	Options		
	Yes	No	DK
Q1. Have you heard of the term organ donation?	240 (95.6%)	3 (1.2%)	8 (3.2%)
Q2. Have you heard of the term organ transplantation?	240 (95.6%)	3 (1.2%)	8 (3.2%)
Q3. Is there an age limit for organ and tissue donation?	102 (40.6%)	120 (47.8%)	29 (11.6%)
Q4. Are you familiar with the diagnosis of brain death?	123 (49.0%)	60 (23.9%)	68 (27.1%)
Q5. To your knowledge, is brain death equivalent to irreversible death?	120 (47.8%)	34 (13.5%)	97 (38.6%)

**Table 3:** Sociodemographic variables and willingness to donate.

Socio- Demographic Variable	Question														
	Q6. Would You Be Willing to Donate Organs after Death?			Q7. Would You Be Willing to Donate Tissues after Death?			Q8. Would You Be Willing during Your Lifetime to Donate Organs after Death to a Family Member in Need?			Q9. Would You Donate a Kidney to a Family Member in Need as a Living Donor?			Q10. Would You Agree to Receive an Organ from a Deceased Donor in a Life-Threatening Condition?		
	Yes	No	DK	Yes	No	DK	Yes	No	DK	Yes	No	DK	Yes	No	DK
Gender															
Male	29 50.9%	7 12.3%	21 36.8%	29 50.9%	7 12.3%	21 36.8%	37 64.9%	7 12.3%	13 22.8%	51 89.5%	2 3.5%	4 7.0%	35 61.4%	7 12.3%	15 26.3%
Female	76 39.4%	47 24.4%	70 36.3%	82 42.5%	38 19.7%	73 37.8%	98 50.8%	23 11.9%	72 37.3%	153 79.3%	15 7.8%	25 13.0%	95 49.2%	29 15.0%	69 35.8%
χ^2^	4.354			2.051			4.354			3.074			2.652		
*p*	0.113			0.359			0.113			0.215			0.266		
Age															
20–40 years	57 36.8%	33 21.3%	65 41.9	63 40.6%	24 15.5%	68 43.9%	82 52.9%	17 11%	56 36.1%	125 80.6%	15 9.7%	15 9.7%	73 47.1%	19 12.3%	63 40.6%
41–60 years	48 50.0%	22 22.9%	26 27.1%	48 50.0%	22 22.9%	26 27.1%	53 55.2%	14 14.6%	29 30.2%	79 82.3%	3 3.1%	14 14.6%	57 59.4%	18 18.8%	21 21.9%
χ^2^	6.157			0.7421			1.300			4.804			9.662		
*p*	0.046 *			0.024 *			0.522			0.091			0.008 *		
Education															
ISCED 3–4	18 26.1%	29 42.0%	22 31.8%	24 34.8%	29 42.0%	26 27.1%	25 36.2%	19 27.5%	25 36.2%	52 75.4%	6 8.7%	11 15.9%	26 37.7%	20 29.0%	23 33.3%
ISCED 5–8	87 47.8%	26 14.3%	69 37.9%	87 47.8%	17 9.3%	78 42.9%	110 60.4%	12 6.6%	60 33.0%	152 83.5%	12 6.6%	18 9.9%	104 57.1%	17 9.3%	61 33.5%
χ^2^	23.715			36.257			23.376			2.304			16.758		
*p*	<0.001 *			<0.001 *			<0.001 *			0.316			<0.001 *		
Religion															
Orthodox	71 36.4%	52 26.7%	72 36.9%	77 39.5%	43 22.1%	75 38.5%	101 51.8%	29 14.9%	65 33.3%	158 81.0%	14 7.2%	23 11.8%	88 45.1%	30 15.4%	77 39.5%
Catholic	8 53.3%	/	7 46.7%	8 53.3%	/	7 46.7%	14 93.3%	/	1 6.7%	12 80%	1 6.7%	2 13.3%	9 60.0%	/	6 40.0%
Protestant	26 63.4%	3 7.3%	12 39.3%	26 63.4%	3 7.3%	12 29.3%	20 48.8%	2 4.9%	19 46.3%	34 82.9%	3 7.3%	4 9.8%	33 80.5%	7 17.1%	1 2.4%
χ^2^	16.719			12.748			14.584			0.190			24.901		
*p*	0.002 *			0.013 *			0.006 *			0.996			<0.001 *		

**Table 4:** Attitudes toward organ donation.

Item	Strongly Disagree	Somewhat Disagree	Neither Agree Nor Disagree	Somewhat Agree	Strongly Agree
Q11. I consider organ donation to be an altruistic gesture.	17 6.8%	46 18.3%	32 12.7%	21 8.4%	135 53.8%
Q12. I like the idea of being able to help someone by donating organs after brain death.	8 3.2%	39 15.5%	41 16.3%	34 13.5%	129 51.4%
Q13. I believe it is a moral duty to accept organ donation if you agree to receive organs.	8 3.2%	53 21.1%	46 18.3%	21 12.4%	113 45.0%
Q14. My choice to become a donor reflects what my family feels I want.	7 2.8%	51 20.3%	73 29.1%	10 4.0%	110 43.8%
Q15. I am afraid of “not being dead” in the case of an organ procurement procedure.	84 33.5%	71 28.3%	73 29.1%	6 2.4%	17 6.8%
Q16. I consider it extremely important that my body be buried “whole.”	85 33.9%	65 25.9%	52 20.7%	16 6.4%	33 13.1%
Q17. Everyone should automatically be considered a potential donor.	29 11.6%	87 24.7%	60 23.9%	22 8.8%	53 31.1%
Q18. After age 18, each individual should decide (legally register) whether or not they wish to become a potential organ donor.	14 5.6%	54 21.5%	49 19.5%	16 6.0%	119 47.4%
Q19. Brain death patients who have not had the opportunity to express their choice during their lifetime should automatically be considered potential donors.	29 11.6%	83 33.1%	71 28.3%	8 3.2%	60 23.9%
Q20. The family has the right to accept or refuse to donate organs in the case of brain dead persons who, during their lifetime, did not have the opportunity to express their choice.	31 12.4%	29 11.6%	36 14.3%	13 5.2%	142 56.6%

**Table 5:** Attitudes toward organ donation mean scores and scale reliability.

Item	Min.	Max.	Mean	SD	Actual Cronbach α 0.708 *
					If Item Were Deleted
Q12. I like the idea of being able to help someone by donating organs after brain death.	1	5	3.94	1.261	0.647
Q11. I consider organ donation to be an altruistic gesture.	1	5	3.84	1.411	0.659
Q20. The family has the right to accept or refuse to donate organs in the case of brain dead persons who, during their lifetime, did not have the opportunity to express their choice.	1	5	3.82	1.503	0.653
Q13. I believe it is a moral duty to accept organ donation if you agree to receive organs.	1	5	3.75	1.307	0.659
Q18. After age 18, each individual should decide (legally register) whether or not they wish to become a potential organ donor.	1	5	3.68	1.392	0.630
Q14. My choice to become a donor reflects what my family feels I want.	1	5	3.66	1.297	0.636
Q19. Brain death patients who have not had the opportunity to express their choice during their lifetime should automatically be considered potential donors.	1	5	2.95	1.336	0.651
Q17. Everyone should automatically be considered a potential donor.	1	5	2.93	1.320	0.666
Q16. I consider it extremely important that my body be buried “whole.”	1	5	2.39	1.356	0.811
Q15. I am afraid of “not being dead” in the case of an organ procurement procedure.	1	5	2.21	1.137	0.756

* Cronbach α value is considered acceptable (0.8 > α ≥ 0.7).

**Table 6:** Attitudes toward organ donation as predictors of willingness to donate.

Item	β	Std. Error	Wald Chi-Square Test	OR (95 CI)	*p*
Q11. I consider organ donation to be an altruistic gesture.	0.282	0.166	2.901	1.1326 0.958–1.835	0.089
Q12. I like the idea of being able to help someone by donating organs after brain death.	0.537	0.239	5.035	1.711 1.070–2.734	0.025 *
Q13. I believe it is a moral duty to accept organ donation if you agree to receive organs.	−0.259	0.217	1.428	0.772 0.505–1.180	0.232
Q14. My choice to become a donor reflects what my family feels I want.	0.458	0.239	3.665	1.581 0.989–2.526	0.056
Q15. I am afraid of “not being dead” in the case of an organ procurement procedure.	−0.038	0.195	0.039	0.962 0.656–1.411	0.844
Q16. I consider it extremely important that my body be buried “whole.”	−0.979	0.232	17.828	0.376 0.238–0.0592	<0.001 *
Q17. Everyone should automatically be considered a potential donor.	0.789	0.226	12.188	2.202 1.412–3.429	<0.001 *
Q18. After age 18, each individual should decide (legally register) whether or not they wish to become a potential organ donor.	−0.758	0.253	8.963	0.468 0.285–0.770	0.003 *
Q19. Brain death patients who have not had the opportunity to express their choice during their lifetime should automatically be considered potential donors.	0.497	0.214	5.406	1.645 1.081–2.501	0.020 *
Q20. The family has the right to accept or refuse to donate organs in the case of brain dead persons who, during their lifetime, did not have the opportunity to express their choice.	−0.003	0.194	0.000	0.997 0.682–1.458	0.990

* *p* < 0.05 statistically significant.

## Data Availability

The data presented in this study are available on request from the corresponding author.
